# Surgical and Endoscopic Resection of Duodenal Neuroendocrine Tumors Have Similar Disease-Specific Survival Outcome

**DOI:** 10.1007/s11605-023-05800-y

**Published:** 2023-08-08

**Authors:** Sarah Mirzaie, Joon Y. Park, Michael A. Mederos, Mark D. Girgis

**Affiliations:** 1 David Geffen School of Medicine, University of California at Los Angeles, Los Angeles, CA USA; 2 Department of Surgery, David Geffen School of Medicine, University of California at Los Angeles, Los Angeles, CA USA

**Keywords:** Duodenal neuroendocrine tumor, Tumor size, NCCN, ENETS, SEER

## Abstract

**Background:**

Duodenal neuroendocrine tumors (dNETs) are rare, and their management is not well-defined. National Comprehensive Cancer Network (NCCN) guidelines recommend surgical resection of large dNETs (> 2 cm) and endoscopic resection of small tumors (< 2 cm). We compared the survival outcomes between surgical and endoscopic resection in various dNET sizes.

**Methods:**

A retrospective cohort study was conducted using patient data from Surveillance, Epidemiology, and End Results Program (SEER) database. Variables analyzed included age, tumor size, grade, stage, and lymph node status. Disease-specific survival (DSS) was compared for endoscopic and surgical groups in dNET size strata: 0–0.5, 0.5–1, 1–2, 2–3, and > 3 cm. Kaplan–Meier and multivariable Cox proportional hazards models were used for survival analysis.

**Results:**

The study included 465 patients, with 124 (26.7%) undergoing surgical resection. The average age was 61.9 years, and tumor sizes ranged from 0.1 to 10.5 cm. Endoscopic resection had 40.5% of tumors between 0 and 0.5 cm, while surgery had only 21% (*p* < 0.001). In the surgical cohort, 79.8% had grade 1 tumors compared to 88.3% in the endoscopy group (*P* = 0.024). Among surgically resected cases, 48.4% (60 patients) had lymph node involvement. Age, tumor size, grade, and stage did not significantly predict survival after surgical resection. Stratified by tumor size, no difference in DSS was observed between surgery and endoscopy groups.

**Conclusions:**

Endoscopic resection demonstrated similar survival outcomes to surgical resection across dNET sizes in this national analysis. Given the risks and the lack of survival benefits for surgery, endoscopic resection may be beneficial for both small and large tumors. Further studies are warranted to validate the current NCCN guidelines.

## Introduction

Duodenal neuroendocrine tumors (dNETs) are rare masses that comprise less than 5% of all neuroendocrine tumors (NETs).^1^ In recent decades, widespread utilization of endoscopic screening methods enhanced our ability to identify dNETs, which seemingly increased the incidence of these tumors.^1^ In general, dNETs tend to have a good prognosis, have small sizes, and are less likely to metastasize; therefore, tumor resection is often curative for localized dNETs.^2^ Endoscopic vs surgical resection of dNETs is often selected based on patient characteristics such as age, comorbid status, and surgical history, as well as tumor characteristics including tumor size, grade, and stage.^3–5^

Tumor resection can be done through either a surgical or endoscopic approach. The National Comprehensive Cancer Network (NCCN) has a proposed set of guidelines for care and management of gastrointestinal NETs of different sizes and locations.^6^ Although there is robust evidence for clinical approach to gastric, ileal, and rectal NETs, due to the rarity of dNETs and a paucity of data on treatment outcomes for these tumors, recommendations for dNETs management are less clearly defined. NCCN recommends surgical resection for dNETs larger than 2 cm (cm) and endoscopic resection only for tumors smaller than 2 cm.^6^ European Neuroendocrine Tumor Society (ENETS) recommends surgical resection for dNETs smaller than 1 cm if periampullary and for dNETs greater than 2 cm if the patient has positive nodal involvement, while it suggests endoscopic resection for dNETs smaller than 1 cm if not periampullary. However, ENETS makes no standardized recommendations for tumors between 1 and 2 cm.^7^

There is limited evidence in the literature regarding the optimal treatment approach for dNETs with regards to endoscopic vs surgical resection based on primary tumor size. Thus, in this study, we sought to understand the lack of standardization in resection of duodenal NETs by comparing long-term survival outcomes of dNETS by resection type and by size using a large national dataset.

## Materials and Methods

A retrospective cohort study was conducted using The Surveillance, Epidemiology, and End Results Program (SEER) database to identify patients with dNETs. The SEER Program is the largest publicly available cancer database, offering comprehensive data on cancer incidence, survival, and mortality. The program collects data from multiple population-based cancer registries across different states, covering approximately 48% of the US population.

### Data Acquisition

Data were extracted from SEER using SEER*Stat 8.4.0.1 and International Classification of Diseases for Oncology, Third Edition (ICD-O-3) codes. Inclusion criteria were “8240/3: Carcinoid tumor, NOS” histology type, and “C17.0-Duodenum” primary site. Exclusion criteria consisted of patients with tumor types “neuroendocrine carcinoma” and “atypical carcinoid tumor,” unavailable survival information, patients with no intervention and tumor only found on autopsy, unknown tumor size, and unknown metastasis. We decided to only include patients with dNETs listed on SEER from 2010 to 2015 to allow for data maturity and meaningful survival analysis. Demographic and tumor-related data were collected for each patient including age, tumor primary site, histologic type, grade, stage, tumor size, number of nodes surgically resected, number of nodes positive for cancer, method of tumor removal including endoscopic or surgical resection, disease-specific survival (DSS) in months, and vital status. The SEER database surgery codes were used based on the American College of Surgeons Commission on Cancer’s Facility Oncology Registry Data System, with supplementary annotations from the SEER Program Coding and Staging Manual.^8^ Patients not undergoing any procedures were excluded, and surgery codes were categorized as endoscopic resection or surgical resection. Furthermore, the status of lymph node resection was also considered when assessing the resection approach. Based on the AJCC TNM staging, N0 was defined as dNETs with no positive nodes detected, N1 as tumor with any nodal involvement, and Nx patients who had endoscopic tumor resection without node removal.^9^ In addition, M0 was defined as tumor with no metastasis, and M1 as tumor with distant metastasis.^9^

### Statistical Analysis

Tumor sizes were stratified into 5 groups—(0–0.5), (0.5–1), (1–2), (2–3), and (> 3) centimeters (cm). In the surgery group, DSS of various tumor size groups was analyzed utilizing Kaplan–Meier (KM) curves through a log-rank method. In addition, to assess the relationship of tumor size with lymph node involvement in the surgery group, the rate of node positivity was calculated for each tumor size stratification as a percentage of patients with positive lymph nodes found on pathology. The impact of size on the rate of node positivity was assessed using chi-square. Univariate Cox analysis was used to identify the variables that may be associated with DSS in the surgical group. Multivariable Cox proportional hazards models were constructed using clinically significant variables to investigate their effect on DSS in the surgical group. Finally, Kaplan–Meier curves were utilized to compare DSS between the surgery group and endoscopic group through comparing N0, N1, and Nx. The *p* values below 0.05 were considered significant. Data analyses were conducted using IBM SPSS Version 26 (IBM Corp., Armonk, N.Y.).

This study is exempt from IRB approval.

## Results

### Baseline Characteristics of Patients and Duodenal NETs

Four hundred and sixty-five patients with dNETs listed on SEER from 2010 to 2015 were identified. The median (IQR) patient follow-up was 5.5 (4.3–7.5) years. The average age of the cohort was 61.9 years. Tumor sizes ranged from 0.1 to 10.5 cm. A total of 124 (26.7%) of the patients underwent surgical tumor resection, and the remaining patients had endoscopic tumor resection. Compared to the endoscopic group, the surgery cohort had a higher percentage of tumors sized between 2 to 3 cm (*p* < 0.001) and tumors larger than 3 cm (*p* < 0.001), while the endoscopic cohort had a higher percentage of tumors smaller than 0.5 cm (*p* < 0.001) and between 0.5 to 1 cm (*p* = 0.015). Among the surgery group, 60 (48.4%) had positive lymph nodes. Ninety-nine (79.8%) of the surgical cohort had histologically low-grade tumors, and 117 (94.4%) had no metastasis at the time of resection (Table 1).

**Table 1:** Patient and tumor characteristics

	Surgery cohort (*n* = 124)	Endoscopy cohort (*n* = 341)	Comparison
Disease-specific survival, median (IQR) months	55.0 (19.0–111.0)	69.0 (55.0–88.0)	
0 to 0.5	58.5 (29.0–111.8)	66.0 (54.0–86.0)	
0.5 to 1	76.0 (40.0–127.0)	70.0 (55.3–88.8)	
1 to 2	58.0 (19.0–127.3)	72.5 (55.8–91.0)	
2 to 3	36.0 (11.0–94.0)	87.0 (69.5–112.3)	
> 3	18.5 (5.3–63.5)	55.0 (50.0–79.0)	
Age at procedure, mean (SD) years	60.7 (11.1)	62.8 (11.8)	0.289
Tumor size cm, *n* (%)			
0 to 0.5	26 (21.0)	138 (40.5)	< 0.001*
0.5 to 1	31 (25.0)	128 (37.5)	0.015*
1 to 2	30 (24.2)	56 (16.4)	0.060
2 to 3	21 (16.9)	8 (2.3)	< 0.001*
> 3	16 (12.9)	11 (3.2)	< 0.001*
Tumor grade, *n* (%)			
1	99 (79.8)	301 (88.3)	0.024*
2	23 (18.5)	40 (11.7)	0.066
3	2 (1.6)	0	0.071
Tumor stage, *n* (%)			
N0	64 (51.6)	-	
N1	60 (48.4)	-	
M0	117 (94.4)	341 (100.0)	< 0.001*
M1	7 (5.6)	0	< 0.001*

Abbreviations: *SD*, standard deviation; *IQR*, interquartile range

### Survival Outcomes for Surgery Group

**Fig. 1:**
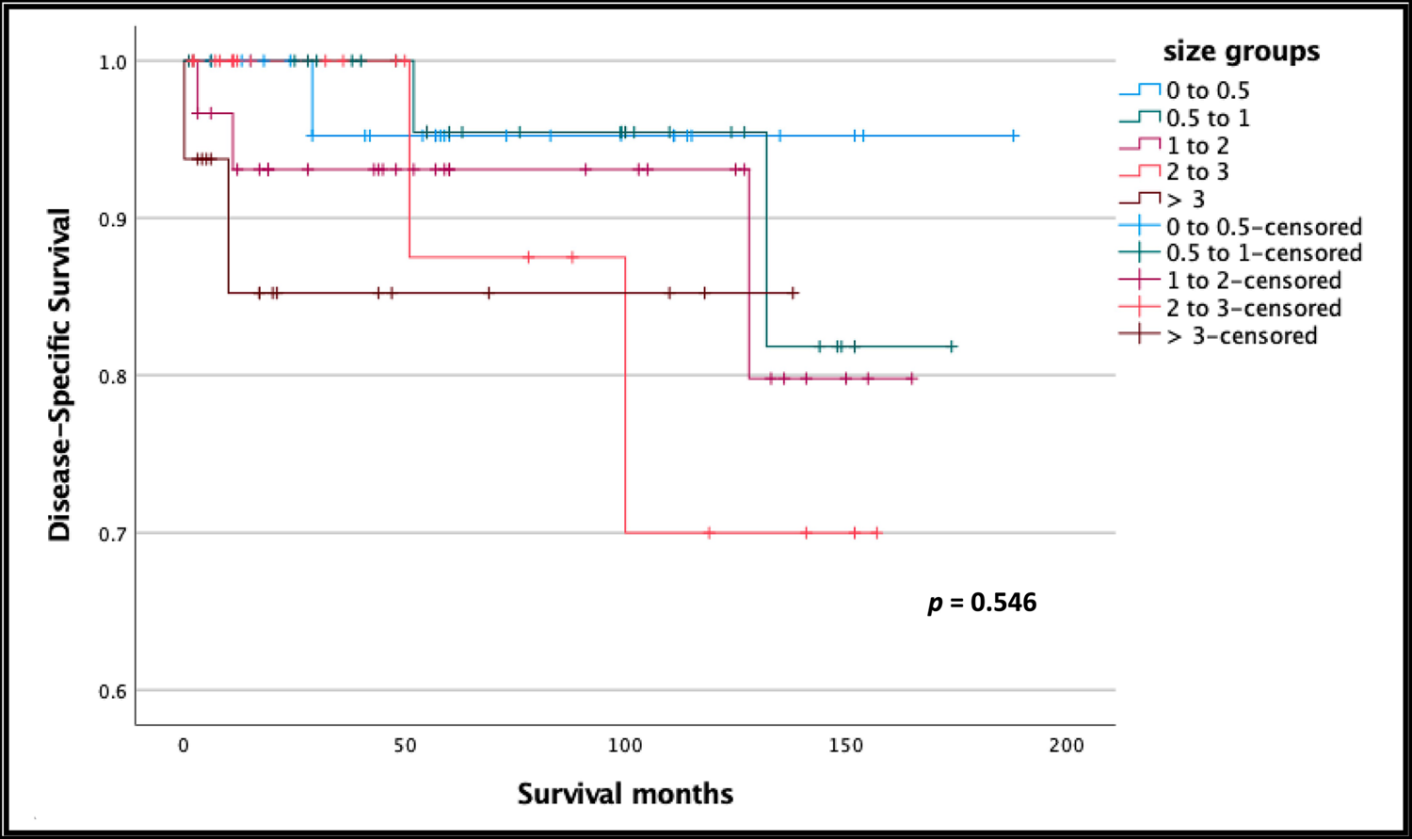
Kaplan–Meier survival curves comparing DSS among patients stratified by tumor size, who underwent surgical tumor resection: (0–0.5), (0.5–1), (1–2), (2–3), and (> 3) cm

**Fig. 2:**
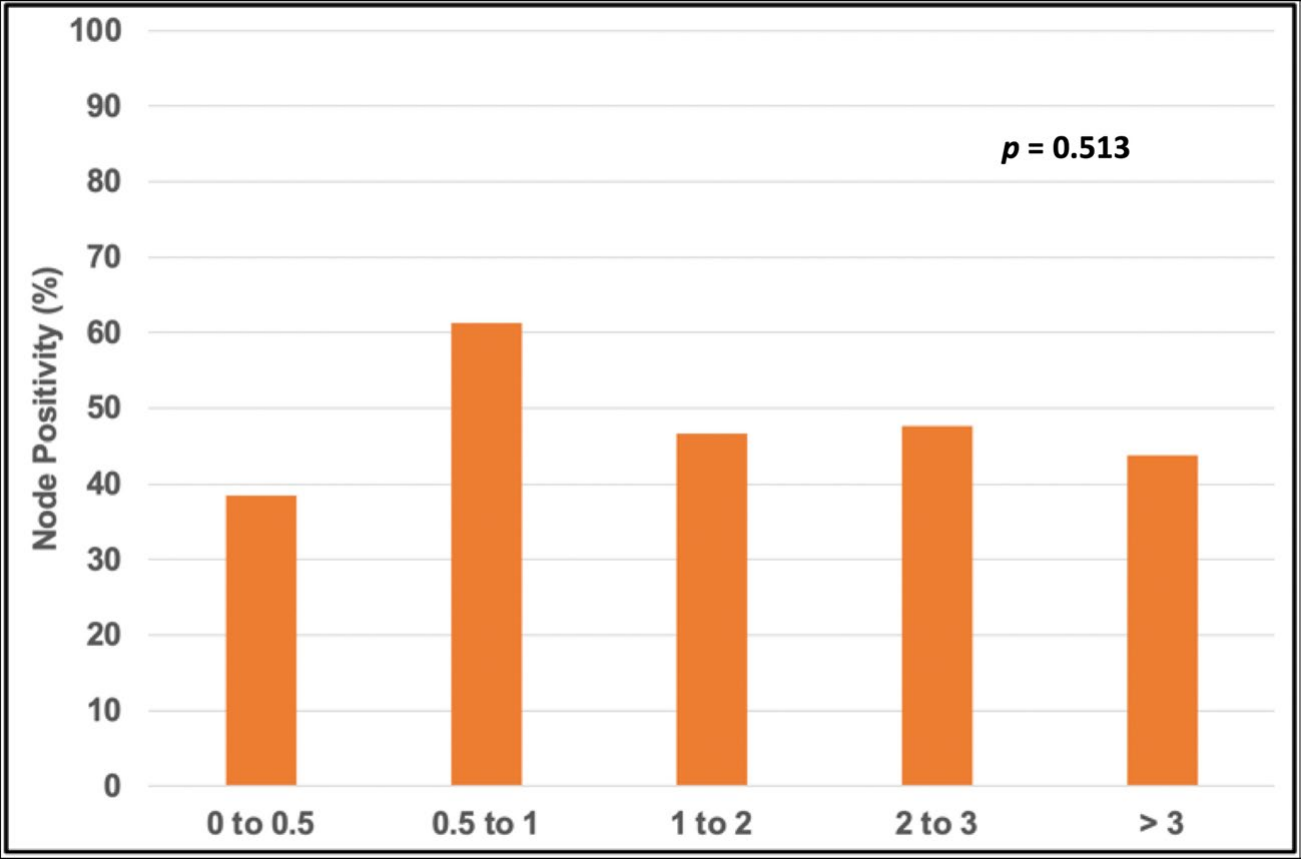
The percentage of node positivity for each size group

Among the patients who underwent surgical tumor resection, tumor size groups—(0–0.5), (0.5–1), (1–2), (2–3), (> 3)—were found to have similar DSS (Fig. 1). In the surgical group, the percentage of node positivity was not associated with size (Fig. 2).

On univariate analysis, age, histologically advanced tumor grade, and nodal involvement were not associated with DSS in patients who underwent surgery. At all size ranges of dNETs, patients had comparable survival after surgical resection regardless of nodal status, tumor grade, or tumor size (Table 2). The hazard ratio for tumor size groups—(0–0.5), (0.5–1), (1–2), (2–3), and (> 3)—and DSS was not statistically significant. In addition, tumor grades 1, 2, and 3, as well as nodal involvement, and distant metastasis did not have a significant hazard ratio with DSS.

**Table 2:** Univariate analysis

	HR (95%)	*p*
Age	0.957 (0.909–1.008)	0.098
Tumor size (cm)		
0 to 0.5	0.390 (0.049–3.083)	0.372
0.5 to 1	0.597 (0.126–2.822)	0.515
1 to 2	1.206 (0.311–4.672)	0.787
2 to 3	1.524 (0.323–7.200)	0.595
> 3	2.902 (0.605–13.927)	0.183
Tumor grade		
1	0.553 (0.154–1.989)	0.364
2	2.210 (0.620–7.880)	0.222
3	-	-
Tumor stage		
N1	5.807 (0.724–46.600)	0.098
M1	0.045 (0–2707.24)	0.580

Subsequently, to assess the predictive value of clinically significant variables in determining survival after surgical tumor resection, a Cox multivariable regression model was constructed. In this model, age, tumor grade or level of differentiation, tumor size, and node positivity did not predict DSS after surgery (Table 3).

**Table 3:** Cox multivariate regression models predicting survival based on tumor size and nodal metastasis in surgery and endoscopy group

Variables	HR (95%)	*p*
Age	0.951 (0.896–1.010)	0.104
Tumor size (cm)		
0 to 0.5	0.123 (0.010–1.450)	0.096
0.5 to 1	0.174 (0.022–1.367)	0.096
1 to 2	0.601 (0.090–4.002)	0.599
2 to 3	0.476 (0.064–3.538)	0.469
Tumor grade		
2	1.938 (0.478–7.859)	0.354
3	-	-
Tumor stage		
N1	6.597 (0.775–56.125)	0.084
M1	-	-

Abbreviations: *HR*, hazard ratio

### Comparison of Survival Outcomes in Surgery vs Endoscopy

**Fig. 3:**
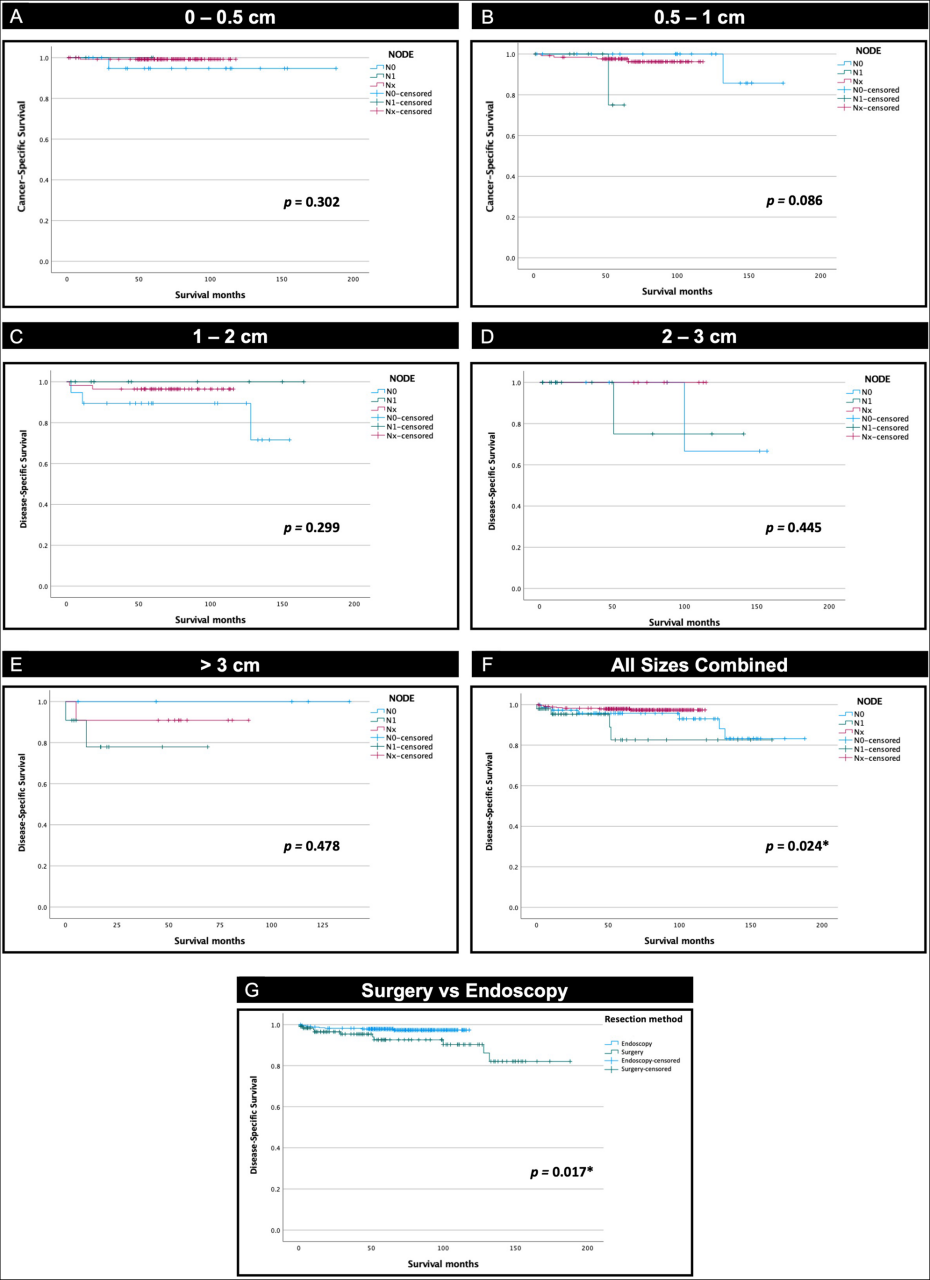
Kaplan–Meier survival curves comparing DSS between N1, N0, and Nx in different size tumors: **A** 0 to 0.5 cm, **B** 0.5 to 1 cm, **C** 1 to 2 cm, **D** 2 to 3 cm, **E** above 3 cm, **F** all sizes combined

At each size range, KM curves were made to compare survival for N1, N0, and Nx. At various size groups—(0–0.5), (0.5–1), (1–2), (2–3), and (> 3)—DSS was similar between N1, N0, and Nx, indicating comparable survival outcomes between surgical and endoscopic tumor resection in this cohort (Fig. 3A–E). When assessing all tumor sizes combined, patients who underwent surgical tumor resection had a mean DSS of 67.3 months and median (IQR; interquartile) of 55.0 (19.0–111.0), which was lower than patients undergoing endoscopic resection with a mean DSS of 71.1 months and median of 69.0 (55.0–88.0). While the mean DSS difference was statistically significant (*p* = 0.017), the 3.8 months survival advantage was not clinically significant (Fig. 3F).

## Discussion

Duodenal NETs are rare masses of the gastrointestinal system. Due to the low incidence and difficulty in diagnosis, dNETs have been studied to a lesser extent compared to other NETs.^1^ Therefore, while there are clear diagnostic recommendations and treatment guidelines for other types of NETs, the clinical approach to dNETs is not as well defined. In this study, we assessed the survival outcome of patients with various size dNETs undergoing endoscopic or surgical tumor resection. Our results indicated that, when controlling for confounding variables, at each tumor size, endoscopic resection had similar survival outcomes to surgical resection.

### Factors Associated with Disease-Specific Survival

Tumor size, grade, and stage, as well as LN positivity, are often utilized to determine disease severity, prognosis, and management plan. Previously, Untch et al. demonstrated that in dNETs, tumor size is associated with recurrence-free survival.^4^ In nonfunctional pancreatic NETs, tumors less or equal to 2 cm with histologic grades of I or II have a low probability of aggressive behavior.^10^ Kim et al. demonstrated that in small intestinal NETs, a higher LN positivity ratio was associated with worse NET cancer-specific survival.^11^

NCCN and ENETS make some recommendations regarding the management of dNETs; however, the role of novel endoscopic approaches in various dNET sizes is still a controversial topic.^6,7^ There is emerging evidence that endoscopic resection of dNETs is as effective in improving patient survival as surgical resection methods.^4,12–14^ In 2021, Tran et al. examined dNETs of different sizes in their cohort (*n* = 104) and found that after adjusting for age, the method of resection had no significant association with survival.^12^ Similarly, Untch et al. (*n* = 75) and Margonis et al. (*n* = 146) suggested that patients undergoing local surgical resection of dNETs had similar survival rates to patients with endoscopic resection.^4,13^ Our findings from a large US national database (*n* = 465) corroborated these recent studies demonstrating that patients undergoing both endoscopic and surgical resection had comparable DSS.

### Lymph Node Positivity and Disease-Specific Survival

Factors associated with lymph node metastasis in dNETs are a topic of debate. ENETS guidelines recommend surgical resection of dNETs larger than 2 cm, in the presence of nodal involvement. Some studies suggest that the size of neuroendocrine tumors may not reliably predict nodal metastasis.^15^ Furthermore, the role of nodal metastasis on survival has also been a controversial topic.^16–18^ While some older studies suggest a role for nodal metastasis on survival of NETs, recently there has been conflicting evidence that suggests otherwise.16–18 The result of our study is in congruence with the recent data as it elucidated that nodal involvement did not predict worse DSS.

Some factors that could be considered regarding lymph node resection include tumor type. Resection of tumor-draining lymph nodes is controversial, particularly in superficial tumors, as extensive lymph node dissection can lead to significant morbidity.^19^ In dNETs, the removal of regional mesenteric lymph nodes has been associated with higher DSS. It is possible that patients would be under-staged if too few lymph nodes are resected, but removing a larger number of lymph nodes may allow for the identification of lymph node metastases that are not clinically detectable.^19^ A study investigated the association of lymph node metastasis on overall survival of dNET patients (*n* = 7613), and their results showed that there was no significant difference in survival between N0 and N1, and even diminished survival for patients who underwent radical lymph node resection compared to local resection.^20^

### Limitations

This study had several limitations that warrant consideration. The distribution of the sample size was such that it limited the granular analysis of tumors larger than 3 cm. However, since in general dNETs tend to be small, then this limitation may have low clinical implications. We collected the patient data from SEER, and due to the limited information on this database, we were unable to account for the effect of other factors on survival such as patient comorbidities, readmissions, and further complications that may have partially explained the differences in survival between endoscopic and surgical cohorts. An important limitation is the lack of comparative analysis between endoscopic and surgical resection in terms of complications, such as the increased risk of perforation associated with larger sizes after endoscopic treatment; therefore, further studies are required to explore this aspect. In addition, the survival difference for surgical resection in larger tumor sizes may have been influenced by selection bias for healthier individuals to undergo endoscopy. Furthermore, an important limitation in the analysis of the endoscopic cohort is their unknown nodal status. Another limitation is the lack of data on SEER regarding the endoscopic cases that required later surgical resection as the database only provides information about the primary procedure. Despite these limitations, this study is clinically important as it investigates the validity of NCCN and ENETS guidelines, which are widely used in clinical practice.

### Clinical Implications

In our study, we found that endoscopic resection of dNETs has a comparable survival outcome to surgical resection at all tumor sizes. This is in contrast to NCCN and ENETS guidelines which do not recommend endoscopic resection for larger tumors. In addition, our results showed that nodal involvement does not significantly impact survival of dNETs of various sizes after surgical resection. Due to the rarity of dNETs, and the relative novelty of endoscopic approaches, there is limited data regarding this topic; however, proper selection of management may have a significant impact on patient care and quality of life. There is robust evidence in the literature that show a higher probability for complications following surgery compared to less invasive endoscopic methods.^21,22^ Because of this, it is pertinent to consider endoscopic resection as a primary approach for dNETs irrespective of tumor size or node positivity, when possible. Further studies need to investigate the validity of current guidelines for dNETs. Additionally, since both endoscopic and surgical resection have similar survival outcomes when correcting for confounders, another important consideration for future studies is direct cost-effectiveness and resources allocation analyses in endoscopic tumor resection compared to a surgical approach.

## Conclusion

When controlling for confounding factors, endoscopic resection of dNETS had similar survival to surgical resection irrespective of size and lymph node status. Given the inherent risk of surgery and lack of survival benefit, it may be beneficial to consider an endoscopic approach whenever technically feasible. Further studies are required to assess the validity of the current NCCN guidelines.

## Data Availability

The data used in this study were collected from the SEER database available online.

## References

[1] Fitzgerald TL, Dennis SO, Kachare SD, Vohra NA, Zervos EE (2015). Increasing incidence of duodenal neuroendocrine tumors: Incidental discovery of indolent disease?. Surgery.

[2] Hoffmann KM, Furukawa M, Jensen RT (2005). Duodenal neuroendocrine tumors: Classification, functional syndromes, diagnosis and medical treatment. Best Pract Res Clin Gastroenterol.

[3] Zhang J, Gold KA, Lin HY, Swisher SG, Xing Y, Lee JJ, Kim ES, William WN (2015). Relationship between tumor size and survival in non–small-cell lung cancer (NSCLC): An analysis of the surveillance, epidemiology, and end results (SEER) registry. J Thorac Oncol.

[4] Untch BR, Bonner KP, Roggin KK, Reidy-Lagunes D, Klimstra DS, Schattner MA, Fong Y, Allen PJ, D’Angelica MI, DeMatteo RP, Jarnagin WR, Kingham TP, Tang LH (2014). Pathologic grade and tumor size are associated with recurrence-free survival in patients with duodenal neuroendocrine tumors. J Gastrointest Surg.

[5] Dogeas E, Cameron JL, Wolfgang CL, Hirose K, Hruban RH, Makary MA, Pawlik TA, Choti MA (2017). Duodenal and ampullary carcinoid tumors: size predicts necessity for lymphadenectomy. J Gastrointest Surg.

[6] Shah MH, Goldner WS, Benson AB, Bergsland E, Blaszkowsky LS, Brock P, Chan J, Das S, Dickson PV, Fanta P, Giordano T, Halfdanarson TR, Halperin D, He J, Heaney A, Heslin MJ, Kandeel F, Kardan A, Khan SA, Kuvshinoff BW, Lieu C, Miller K, Pillarisetty VG, Reidy D, Salgado SA, Shaheen S, Soares HP, Soulen MC, Strosberg JR, Sussman CR, Trikalinos NA, Uboha NA, Vijayvergia N, Wong T, Lynn B, Hochstetler C (2021). Neuroendocrine and adrenal tumors, version 2.2021, NCCN clinical practice guidelines in oncology. J Natl Compr Cancer Netw.

[7] DelleFave G, Kwekkeboom DJ, Van Cutsem E, Rindi G, Kos-Kudla B, Knigge U, Sasano H, Tomassetti P, Salazar R, Ruszniewski P (2012). ENETS consensus guidelines for the management of patients with gastroduodenal neoplasms. Neuroendocrinology.

[8] Wani S, Drahos J, Cook MB, Rastogi A, Bansal A, Yen R, Sharma P, Das A (2014). Comparison of endoscopic therapies and surgical resection in patients with early esophageal cancer: a population-based study. Gastrointest Endosc.

[9] Amin MB, Greene FL, Edge SB, Compton CC, Gershenwald JE, Brookland RK, Meyer L, Gress DM, Byrd DR, Winchester DP (2017). The Eighth Edition AJCC cancer staging manual: Continuing to build a bridge from a population-based to a more “personalized” approach to cancer staging. CA Cancer J Clin.

[10] Fathi AH, Romanyshyn J, Barati M, Choudhury U, Chen A, Sosa JA (2020). Predicting aggressive behavior in nonfunctional pancreatic neuroendocrine tumors with emphasis on tumor size significance and survival trends: a population-based analysis of 1787 patients. Am Surg.

[11] Kim MK, Warner RRP, Ward SC, Harpaz N, Roayaie S, Schwartz ME, Itzkowitz S, Wisnivesky J (2015). Prognostic significance of lymph node metastases in small intestinal neuroendocrine tumors. Neuroendocrinology.

[12] Tran CG, Sherman SK, Suraju MO, Nayyar A, Gerke H, El Abiad RG, Chandrasekharan C, Ear PH, O’Dorisio TM, Dillon JS, Bellizzi AM, Howe JR (2022). Management of duodenal neuroendocrine tumors: surgical versus endoscopic mucosal resection. Ann Surg Oncol.

[13] Margonis GA, Samaha M, Kim Y, Postlewait LM, Maithel SK, Poultsides GA, Tran T, Gamblin TC, Berger NG, Pawlik TM (2016). A multi-institutional analysis of duodenal neuroendocrine tumors: Tumor biology rather than extent of resection to dictate prognosis. J Clin Oncol.

[14] Kim GH, Il Kim J, Jeon SW, Moon JS, Chung I-K, Jee S-R, Kim HU, Seo GS, Baik GH, Lee YC, Research TKC of H and UG (2014). Endoscopic resection for duodenal carcinoid tumors: A multicenter, retrospective study. J Gastroenterol Hepatol.

[15] Mullen JT, Wang H, Yao JC, Lee JH, Perrier ND, Pisters PWT, Lee JE, Evans DB (2005). Carcinoid tumors of the duodenum. Surgery.

[16] Jiang J, Park J, Kim S, Daan A, Donahue T, Girgis MD (2021). Surgical resection of high-grade nonfunctional pancreatic neuroendocrine carcinoma is associated with improved survival. J Surg Oncol.

[17] Sohn B, Kwon Y, Ryoo S-B, Song I, Kwon Y-H, Lee DW, Moon SH, Park JW, Jeong S-Y, Park KJ (2017). Predictive factors for lymph node metastasis and prognostic factors for survival in rectal neuroendocrine tumors. J Gastrointest Surg.

[18] Conrad C, Kutlu OC, Dasari A, Chan JA, Vauthey J-N, Adams DB, Kim M, Fleming JB, Katz MHG, Lee JE (2016). Prognostic value of lymph node status and extent of lymphadenectomy in pancreatic neuroendocrine tumors confined to and extending beyond the pancreas. J Gastrointest Surg.

[19] Zaidi MY, Lopez-Aguiar AG, Dillhoff M, Beal E, Poultsides G, Makris E, Rocha F, Crown A, Idrees K, Marincola Smith P, Nathan H, Beems M, Abbott D, Barrett JR, Fields RC, Davidson J, Cardona K, Maithel SK (2019). Prognostic role of lymph node positivity and number of lymph nodes needed for accurately staging small-bowel neuroendocrine tumors. JAMA Surg.

[20] Fujii Y, Tzeng C-W, Chiang Y-J, Halperin DM, Dasari A, Kim MP, Katz MHG, Lee JE, Ikoma N (2021). Incidence of lymph node metastases and impact of radical surgery for duodenal neuroendocrine tumors. J Surg Res.

[21] Smith JK, Ng SC, Hill JS, Simons JP, Arous EJ, Shah SA, Tseng JF, McDade TP (2010). Complications after pancreatectomy for neuroendocrine tumors: a national study. J Surg Res.

[22] Kaçmaz E, Chen JW, Tanis PJ, Nieveen van Dijkum EJM, Engelsman AF (2021). Postoperative morbidity and mortality after surgical resection of small bowel neuroendocrine neoplasms: A systematic review and meta-analysis. J Neuroendocrinol.

